# Data set of production of castor oil and characterization of cotton and castor mixed seed oil additives with diesel fuel

**DOI:** 10.1016/j.dib.2024.110210

**Published:** 2024-02-22

**Authors:** Hailegebrel Zewdie Woldetensay, Dinku Seyoum Zeleke, Getachew Shunki Tibba

**Affiliations:** Department of Mechanical Engineering. Addis Ababa Science and Technology University, Sustainable Energy Center, Addis Ababa, Ethiopia 16417, Ethiopia

**Keywords:** Diesel fuel, Bio additives, Caster seed oil, Cottonseed oil, Fatty acid methyl esters, Gas chromatography

## Abstract

An important energy source for industry and transportation is diesel fuel. Nonetheless, the use of diesel fuel has been connected to a number of environmental problems, such as climate change and air pollution. The purpose of this data set research is to extract oil from castor seeds and cottonseeds using a mechanical press method to use as lubricant. The oil is refined to remove impurities and improve its quality once it is extracted. The next step was determining the fatty acid content of castor oil, cottonseed oil, and cottonseed oil (50%) mixed with castor seed oil (50%) using gas chromatography (Agilent 7890B) with a mass spectroscopy detector (Agilent 5977A MSD, USA) and the European standard (EN 14103:2011). There were thirteen (13) significant methyl esters of fatty acids found. Furthermore, to make sure they met the specifications needed for dependable engine operation, the reference diesel and the diesel fuel with 0.25%, 0.50%, 0.75%, and 1% bio additives (mixed cottonseed oil, 50%; and caster seed oil, 50%) were characterized. It was subsequently determined that the physicochemical properties, including density, kinematic viscosity, calorific value, and total sulfur, complied with stated ASTM requirements. The results of the investigation showed that the fatty acid profile of combined cotton and caster has the advantage of both oils' quality, with all of its physicochemical properties falling within the ASTM recommendations for diesel fuel. In order to improve lubricity in diesel engines, 50% of caster seed oil and 50% of mixed cottonseed oil were used as bio-additives.

Specifications Table

**Table:** 

Subject	*Bioenergy*
Specific subject area	*Biodiesel and engine tests*
Data format	*Primary analyzed data*
Type of data	*Figures and Tables*
Data collection	*Data are collected and produced using a mechanical presses and then analyzed using Bomb calorimeter, gas chromatography-mass spectrometry (GC MS),Mechanical press machine*
Data source location	*At Addis Ababa Science and Technology University on January 19/2024 on Mendeley data set*
Data accessibility	Repository name: Castor Oil and Characterization of Cotton and Castor Mixed Seed Oil Additives with Diesel Fuel https://data.mendeley.com/drafts/dx4tfs8t6r

## 1 Value of the Data

• Combining cottonseed oil and caster seed oil improves their respective advantages when used as additives in diesel engines that run on low sulfur diesel fuel. Both oils have unique fatty acid profiles. While increasing the additives from 0.25% to 0.50%, the qualities remain similar to diesel fuel with the added advantages of high lubricity and sulfur reduction from diesel fuel.
• 0.0001.0.0002 and 0.0003 at 0.75% and 1%, respectively reduce Diesel fuel's sulfur level, with a notable impact on emissions and environmental preservation. Therefore, diesel engines can benefit from the addition of blended cottonseed and caster seed oils.
• This study set out to determine the characteristics of bio-additives derived from mixed caster and cottonseed oils. Based on the fuel's qualitative characteristics and its satisfactory compliance with ASTM D 6751 criteria, diesel fuel containing a blend of caster and cottonseed oil additives can be used as an alternative to conventional diesel fuel.
• Africa is home to a vast amount of cottonseed and castor seed, both of which have several uses in the agricultural, pharmaceutical, cosmetic, paint, detergent, and as engine lubricant. They also possess antioxidant and anti-allergic properties. This blended biodiesel is therefore an economical choice for lubricating motors. Since diesel is blended with less than 1% of castor oil and cottonseed to act as an engine lubricant.

## 2 Background

Diesel engines will meet around half of the world's energy demand by 2040, continuing to be the top contenders in the transportation sector [1]. However, the usage of diesel fuel has been connected to a number of environmental problems, including climate change and air pollution. This is because the high sulfur content of ordinary fossil diesel fuel, which powers engines, leads to a large amount of oxides being produced from the exhaust gas of diesel engines. Acid rain is a result of air pollution caused by harmful oxides, which is detrimental to human health [2]. The primary source of SO2 emissions is the burning of fossil fuels that include significant concentrations of this element. Acid rain is the result of these fuels rehydrating with atmospheric water [[3], [4]]. In addition to having an offensive odor, SOx can produce acid rain when its concentration is too high in the atmosphere. The development of acid rain will significantly lower the survival rate of different plants, as well as have an impact on crop growth and the nutritional value of food for humans [5].

## 3 Data Description

When 5000 g of refined caster seed yielded 1190.5 ml of oil, the released oil proceeded on to the decantation step. Following the extraction of 1190.5 ml of oil, it was found that the oil yield, or oil content, of the seed was 23.78%. As a result, it was shown to be worth less than the earlier conclusions [6]. The low oil content that resulted was caused by the mechanical press's inefficiency. To extract even more oil from the seeds and increase the efficiency of the mechanical press, the leftover cake is subjected to a solvent extraction process after mechanical extraction.

**Fig. 1:**
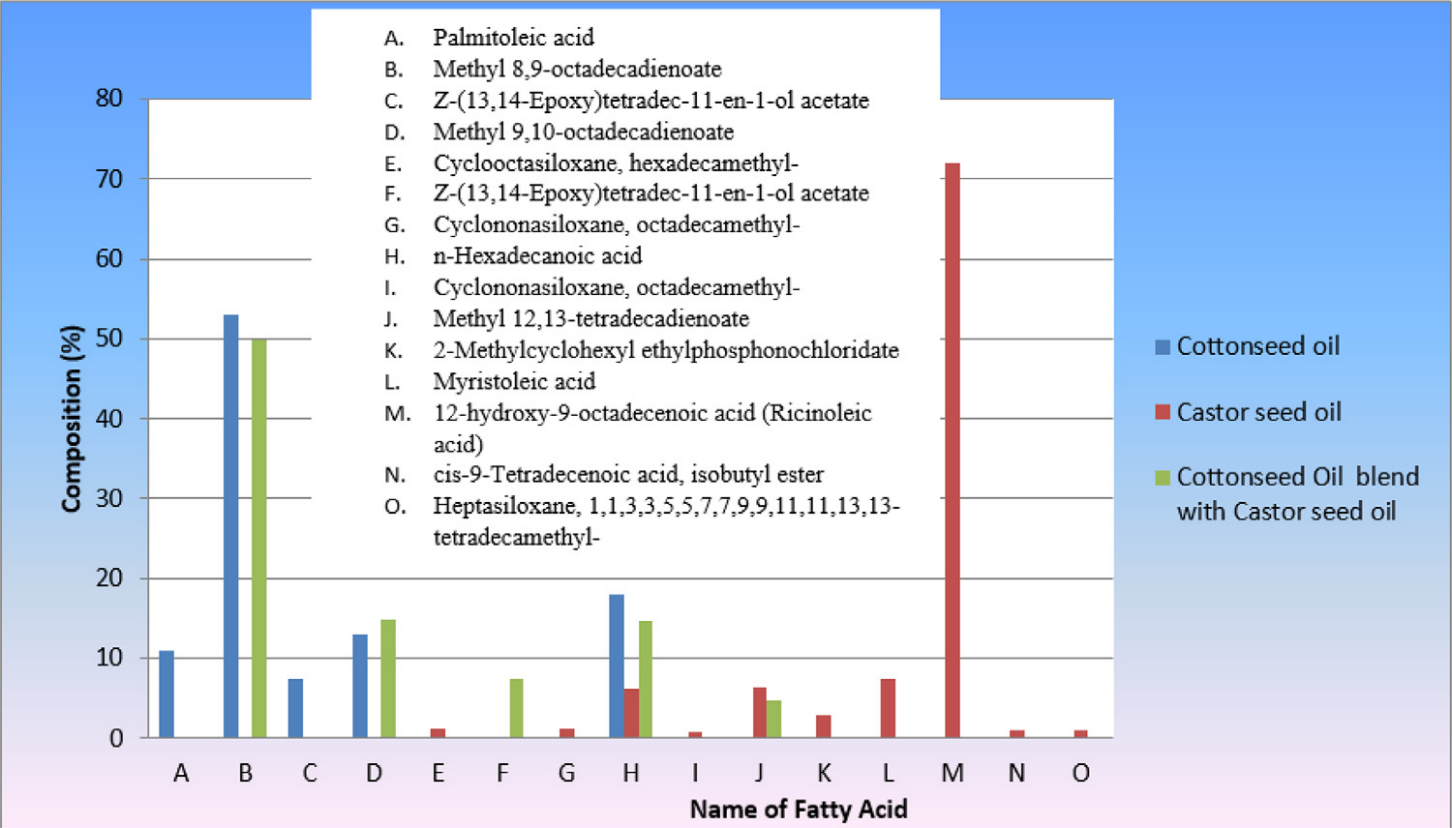
The fatty acid composition of cottonseed oil, caster seed oil and blend of 50% cottonseed oil with 50% caster seed oil.

Determining the composition of oils, together with their fatty acid concentrations and glyceride distribution pattern, is essential because the composition of oils directly affects their physical properties and end-use performance [7]. These days, the composition of fatty acids is the primary attribute of oils and fats. The fatty acid content of castor seed oil, cottonseed oil, and a mixture of 50% castor seed oil and 50% cottonseed oil was measured using gas chromatography. The outcomes are mostly in line with those published in previous studies [2], [3], [4]. The outcomes are illustrated in Table 1. A typical chromatogram for the castor seed oil, cottonseed oil, and cottonseed oil combination is shown in Fig. 1. The primary component of castor oil, ricinoleic acid, is broken down methanolytically to produce methyl ricinoleate, which is shown by the creation of a large peak in methyl ester.

**Table 1:** Fatty acid composition of Cottonseed oil, Castor seed oil and Cottonseed oil blend with Castor seed oil.

Fatty acid name	Chemical structure	Composition					
		Cottonseed oil		Castor seed oil		Cottonseed Oil blend with Castor seed oil	
		RT	(Area.%)	RT	(Area.%)	RT	(Area.%)
Methyl 8,9-octadecadienoate (Linoleic acid,C18:2)	C_19_ H_34_ O_2_	35.69	52.95			26.10	49.90
Z-(13,14-Epoxy) tetradec‑11-en-1-ol acetate	C_16_ H_28_ O_3_					31.29	7.51
Methyl 9,10-octadecadienoate (Methyl linoleate; C18:2)	C_19_ H_34_ O_2_	35.86	13.10			26.25	14.94
Cyclooctasiloxane, hexadecamethyl-	C_16_ H_48_ O_8_ Si_8_			12.85	1.12		
Cyclononasiloxane, octadecamethyl-	C_18_ H_54_ O_9_ Si_9_			15.81	1.15		
n-Hexadecanoic acid(palmitic acid)	C_16_ H_32_ O_2_	30.45	18.08	18.62	6.25	19.56	14.63
Cyclononasiloxane, octadecamethyl-	C_18_ H_54_ O_9_ Si_9_			19.30	0.82		
Methyl 12,13-tetradecadienoate	C_15_ H_26_ O_2_			24.78	6.40	30.77	4.64
2-Methylcyclohexyl ethylphosphonochloridate	C_9_ H_18_ Cl O_2_ P			26.86	2.90		
Myristoleic acid	C_14_ H_26_ O_2_			29.64	7.42		
12‑hydroxy-9-octadecenoic acid (Ricinoleic Acid,18:1-OH)	C_18_ H_34_ O_3_			30.52	71.99		
cis-9-Tetradecenoic acid, isobutyl ester	C_18_ H_34_ O_2_			32.01	0.93		
Heptasiloxane, 1,1,3,3,5,5,7,7,9,9,11,11,13,13-tetradecamethyl-	C_14_ H_44_ O_6_ Si_7_			33.69	0.99

Table 1 summarize the results of the gas chromatography study and provide a qualitative description of the fatty acid content of cottonseed oil, castor bean oil, and cottonseed oil (50%) blended with 50% castor seed oil. The fatty acid profile of the cottonseed oil was as follows: Methyl 8, 9-octadecadienoate (52.9455%), Methyl 9, 10-octadecadienoate (13.00678 %), and n-Hexadecanoic acid (18.08016 %). It was found that the fatty acid profile of cotton seed oil agreed with the results that had previously been published [8].

The fatty acid composition of Castor oil has been determined to include the following: Cyclooctasiloxane, hexadecamethyl-(1.12396%), Cyclononasiloxane, octadecamethyl-(1.15597%), n-Hexadecanoic acid (6.25117%), methyl 12,13-tetradecadienoate (6.40134%), 2-Methylcyclohexyl ethylphosphonochloridate (2.90267%), Myristoleic acid (7.42198%), 12‑hydroxy-9-octadecenoic acid (Ricinoleic acid) (71.9933%), cis-9-Tetradecenoic acid, isobutyl ester (0.93678%), and heptasiloxane, 1,1,3,5,5,7,7,9,9,11,11,13,13,13-tetradecamethyl- (0.99454%). It was found that the cottonseed oil's fatty acid profile agreed with the results that had been previously published [30]. In the 50% blend of Castor seed oil and Cottonseed oil, the following fatty acid profiles were detected: Methyl 12,13-tetradecadienoate (4.64438%), Z-(13,14-Epoxy)tetradec‑11-en-1-ol acetate (7.50599%), Methyl 9,10-octadecadienoate (Methyl linoleate; C18:2) (14.9364%), n-Hexadecanoic acid (palmitic acid) (14.6338%), and Methyl 8,9-octadecadienoate (49.9018%).Cotton seed oil has more polyunsaturated fatty acid known as linoleic acid (methyl 8,9-octadecadienoate) than caster seed oil. Comparatively, caster seed oil has more monounsaturated fatty acids due to the presence of methyl 8, 9-octadecadienoate (Linoleic acid, C18:2). The 50% cottonseed oil and 50% caster seed oil blend has less mono and poly unsaturated fatty acids than both types of oil alone.

The physicochemical properties of every produced fuel were measured in accordance with ASTM recommendations. The diesel fuel that had been blended with 50% cottonseed oil and 50% caster seed oil, or bio-additives, at concentrations of 0.25%, 0.50%, 0.75%, and 1%, as well as reference diesel, were examined to ensure that they fulfilled the requirements needed to ensure optimal engine performance. According to published ASTM standards, physicochemical parameters such as density, kinematic viscosity, calorific value, and total sulfur were determined. The results are presented in Table 2. Diesel's characteristics are found to be similar to those of diesel fuel with blended cottonseed oil (50%) and caster seed oil (50%) bio-additives.

**Table 2:** Physicochemical properties of tested fuel samples.

Property	Test methods	Physicochemical properties of fuel and its additives					References [31]
		Diesel fuel	D99.75% + 0.25% (cotton &Caster seed oil	D99.50% + 0.50% (cotton &Caster seed oil	D99.25% + 0.75% (cotton &Caster seed oil	D99.00% + 1.00% (cotton &Caster seed oil	
Density@15 °C, kg/m^3^	D4052	843.4	843.8	844.2	844.6	845.0	
Density@20 °C, kg/m^3^	D4052	839.9	840.3	840.7	841.1	841.5	
Kinematic viscosity 40 °C mm²/s	D445	3.01	3.02	3.03	3.05	3.06	
Calorific value, MJ/kg	Calculated	45.54	45.53	45.52	45.52	45.50	
Total sulfur, %WT	D4294	0.05	0.05	0.05	0.05	0.05	

**Fig. 2:**
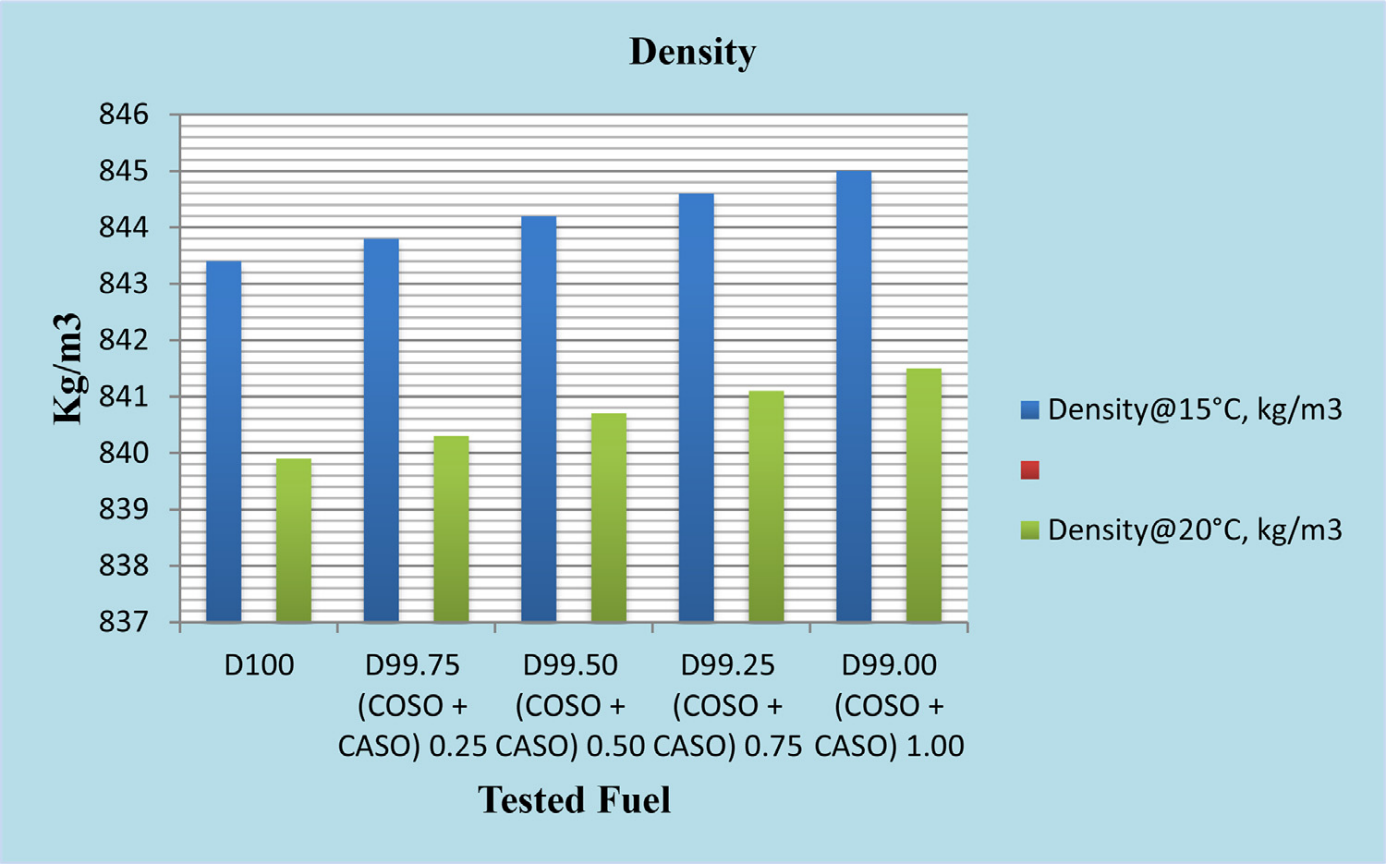
Density at 15 °C, kg/m3 and 20 °C,kg/m3 of diesel fuel with 50% cotton seed oil and 50% caster seed oil blend as additives.

It is imperative to keep density values within reasonable bounds in order to facilitate optimal air-to-fuel ratios for complete combustion. Density is an important fuel characteristic because injection systems, pumps, and injectors need to provide a precisely regulated amount of fuel to ensure optimal combustion. A blend of high-density biodiesel might cause incomplete combustion and particulate matter emissions [9]. Diesel fuel and 50% cotton and 50% caster seed oil additives are found to have kg/m3 values of 843.4 to 845.0 at density@15 °C; at density at 20 °C, the same values are determined to be 839.9 to 841.5. As shown in Fig. 2, when the additions of caster seed oil and mixed cotton were increased from 0.25% to 1%. Although the fuel blend containing cotton and caster seed oils has increased in density, it remains within ASTM standard limits.

**Fig. 3:**
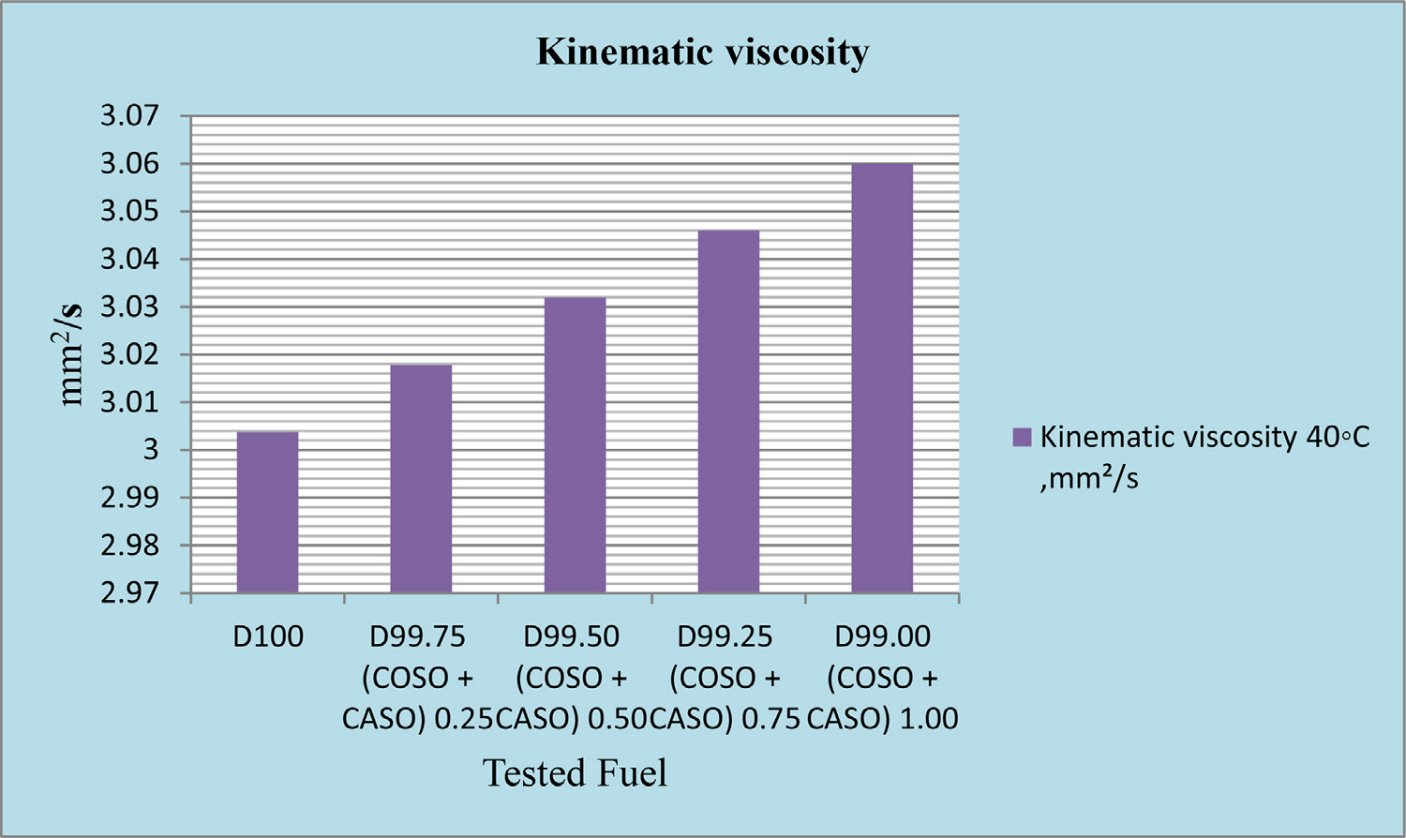
Kinematic viscosity 40 °C mm²/s of diesel fuel with 50% cottonseed oil and 50% caster seed oil blend as additives.

The most important property of any fuel is its viscosity, which is a measure of the material's capacity for flow. As a result, it influences the operation of fuel injection equipment and spray atomization, particularly at low temperatures when the increase in viscosity decreases fuel fluidity. Increased viscosity lowers combustion efficiency through ineffective fuel injection atomization, which results in power losses [10]. The maximum allowable limit is (1.9–6.0 mm2/s) according to the ASTM D445 ranges [11]. As illustrated in Fig. 3, the addition of caster seed oil to blended cotton was increased from 0.25% to 1%. The mixture of cotton and caster seed oil does not exceed the ASTM standard limit, even though there is a rise in kinematic viscosity.

**Fig. 4:**
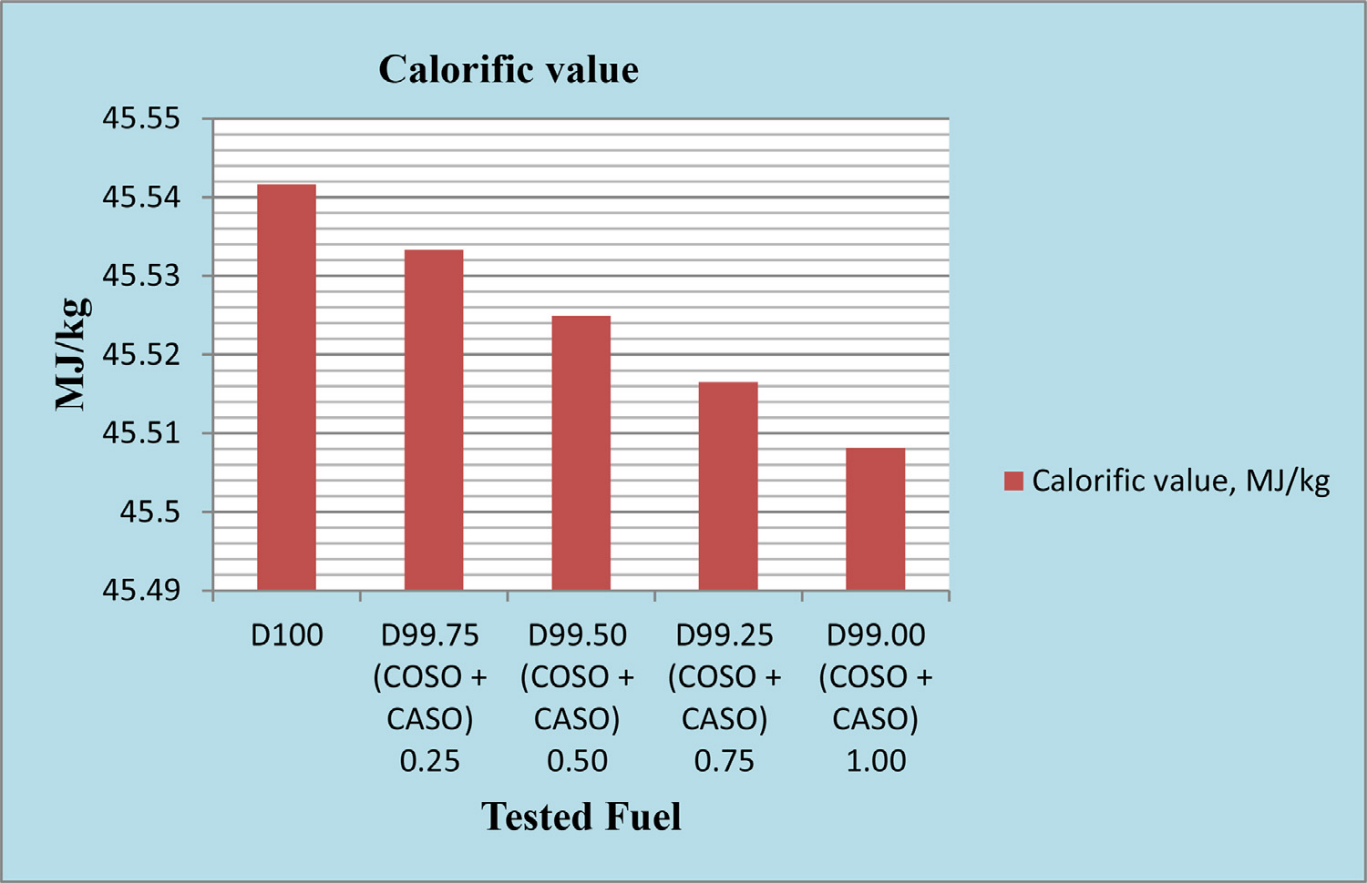
Calorific value, MJ/kg of diesel fuel with 50% cottonseed oil and 50% caster seed oil blend as additives.

The calorific value of diesel engine fuel is a significant factor that influences fuel economy and consumption [11]. Using a bomb calorimeter, it was found that diesel fuel had a higher calorific value than mixed cotton and caster seed oil. The approximately 10% oxygen content of vegetable oil, which has a lower calorific value, causes the calorific value of combined cotton and caster seed oil to decline by 0.02% and 0.07% when the additions are raised from 0.25% to 1%, as illustrated in Fig. 4. When diesel fuel is combined with caster seed and cottonseed oil additives, the reduced calorific valve increases the fuel consumption specifically for the brakes [12]. The total density of the cotton and caster seed oil additives with diesel fuel is larger than that of diesel fuel, though, because the injection pump works on a volume basis. This means that there is more energy released during combustion than there is with diesel. Some studies claim that a slight increase in engine power and moment occurs when the oxygen content of the biodiesel fuel burns in rich flame zones [13].

**Fig. 5:**
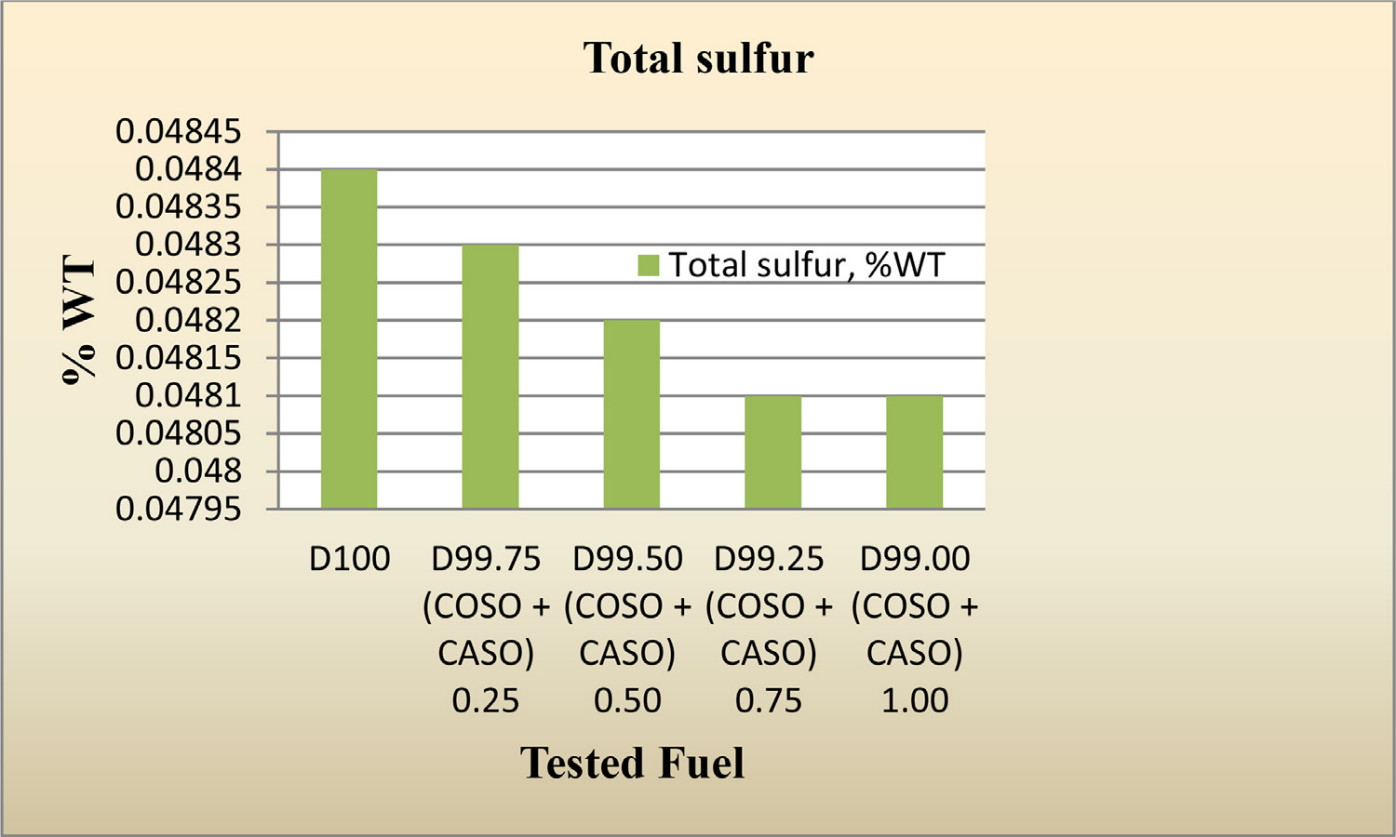
Total sulfur,% WT of diesel fuel with 50% cotton seed oil and 50% caster seed oil blend as additives.

Sulfur dioxide (SOx) emissions are among the most difficult to control and lead to the worst pollution; this is despite efforts to reduce pollutant emissions. Several approaches have been put up, such as the utilization of bio-based fuels or other renewable energy sources. As shown in Fig. 5, the sulfur level of the diesel fuel drops and becomes more environmentally friendly when the amounts of blended cotton and caster seed oil are raised from 0.25% to 1%.

Ultra-low sulfur diesel reduces SOX and particle emissions but retains a low level of lubricity, which could cause wear and friction. For this reason, it is essential to add additional additives to maintain enough lubricity. A possible way for reducing the lubricity loss of diesel fuel is to blend the biodiesel (fatty acid methyl esters) or oxygenated compounds as additives [[14], [15], [16]]. When a protective layer forms on the surfaces of relative moving parts, they can withstand more wear and tear. Through its ability to keep the parts from coming into contact with one another, this film lowers friction and wear. Fuel consumption can be lowered by 1.5–2.5 percent if mechanical friction losses are decreased by 10%.

## 4 Experimental Design, Materials and Methods

### 4.1 Materials

Low-sulfur diesel gasoline was bought for this study from a local Ethiopian petrol station. The Addis Modjo Edible Oil Complex Share Company in Ethiopia was the source of the cottonseed oil acquired. Gossypium arboreum, often known as cotton, is a member of the Malvaceae, or mallow, family. Cottonseed oil (CSO) is a valuable commodity that is recovered from one of the byproducts of cottonseeds and is a major annual fiber crop with significant commercial value. The types of cotton farmed, the locations and seasons in which they are grown, and the extraction techniques employed all affect the oil yield. Ricinus communis L, the botanical name of the castor plant, belongs to the Eurphorbiaceae family and is currently grown and naturalized in all temperate regions of the world. For the production of soaps, lubricants, coatings, and other products, it is generally recognized as having significant economic value. Nowadays, castor oil is recognized as a valuable feedstock for bio refineries because it is non-edible and does not compete with food chains in the production of biofuels as well biochemical, and biopolymers.

### 4.2 Experimental design (Preparation and extraction of caster seed oil)

To obtain a good output from the castor oil production process, castor seeds must go through several preparatory steps. To get rid of unwanted foreign particles, collected seeds are cleansed with tap water. After that, heat is applied to the castor seeds to extract as much moisture as feasible. To extract the oil using mechanical pressing, castor seed was fed into a presser that generates high pressure. Following a single extraction cycle, the machinery was disassembled to remove the solid cake from the unit and collect the oil on a different side. Because the recovered oil also contained some impurities, it was further filtered by running it through a screen.

### 4.3 Methods

The separation method known as gas chromatography is based on the variations in the immiscible phases (mobile and stationary) for which the chemicals in a mixture have varying affinities. A capillary column covered with a stationary phase (liquid or solid) is used to vaporize the sample into the gas phase and separate it into its constituent parts. Solid, gaseous, and liquid materials can all be studied with it. Identifying unknown compounds or pollutants is much easier with the use of mass spectrometry. It calculates the ions produced by the sample in terms of mass to charge ratio. These ions are created by ionisation procedures such as electron impact (EI) or chemical ionisation (CI). Compounds are detected, identified, and quantified using it in accordance with their mass-to-charge ratio.

**Fig. 6:**
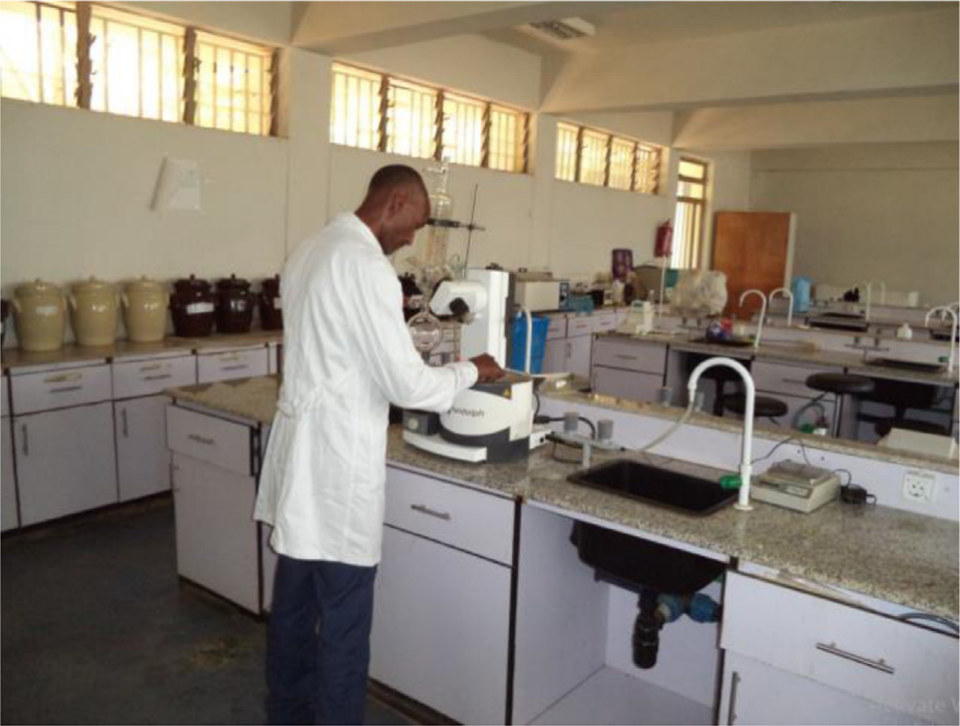
Principal investigator on gas chromatography analysis (Arba Minch University Natural Science laboratory).

Utilizing gas chromatography-mass spectrometry (GC MS), the vegetable oil's fatty acid composition was assessed. Gas chromatographic analysis can only be carried out with a volatile sample. Non-polar and non-volatile compounds are fatty acids. The fatty acids included in the oil were made volatile by derivatization, which was done prior to GC–MS analysis. Methylation is the most widely used method for converting non-volatile fatty acids into volatile methyl esters of fatty acids. Following standard protocol, methylation of fatty acids was performed with a derivatizing reagent of boron trifluoride-methanol (BF-M), the most accepted technique for converting fatty acids into methyl esters of fatty acids. Using gas chromatography (Agilent 7890B) and a mass spectrometer (Agilent 5977A MSD, USA) under European standard (EN 14,103:2011) operating conditions, the FA composition of cottonseed oil, castor oil, and cottonseed oil (50%) mixed with castor seed oil (50%) was ascertained, as shown in Table 3. The components were finally validated by comparing their mass fragmentation patent and retention times with those available in the NIST library of the National Institute of Standards and Technology. The gas chromatography analysis procedure used by the principal author at Arba Minch University to examine the three oils—castor oil, cottonseed oil, and both castor 50% and cottonseed oil 50%—is depicted in Fig. 6.

**Table 3:** Gas chromatography operating conditions.

Operating parameter	Specifications
Column	Hp-88,(30 m x 0.25 mm x 0.20 µm)
Carrier gas	He
Column Flow	1 ml/min
Split ratio	100
Inlet Temperature	250 °c
Transfer line temperature	240 °c
Ion source temperature	230 °c
Oven temperature program	From 50 °c increased by 15 °c/min to 130 °c,by 4 °c/min to 145 °c stays for 13 min, then increased by 5 °c/min to 205 °c and finaly by 10 °c/min to 230 °c

A volume-to-volume breakdown of the production of extra fuel using the same additives is shown in Table 4. Several cotton fuel samples were added to diesel fuel that had been blended with castor seed oil additives (ppm) for this investigation. Diesel fuel (D100) was compared with the fuel samples that were produced. D99.75 was developed by combining diesel and a blend of cotton and castor oil, with 0.25 of the blend used as an additive. The mixture was blended with 0.25% cotton and castor oil and 99.75% diesel (by volume). It was then agitated for 30 min at 700 rpm until it was homogenous.

**Table 4:** Composition of all test fuel with vegetable oil additives volume/volume basis.

Fuel blend	Diesel	vegetable oil additives
D100	100%	0
D99.75 (COSO + CASO) 0.25	99.75%	0.25% (cotton seed oil with caster seed oil blend)
D99.50 (COSO + CASO) 0.50	99.50%	0.50% (cotton seed oil with caster seed oil blend)
D99.25(COSO + CASO) 0.75	99.25%	0.75% (cotton seed oil with caster seed oil blend)
D99 (COSO + CASO) 1	99%	1% (cotton seed oil with caster seed oil blend)

**Fig. 7:**
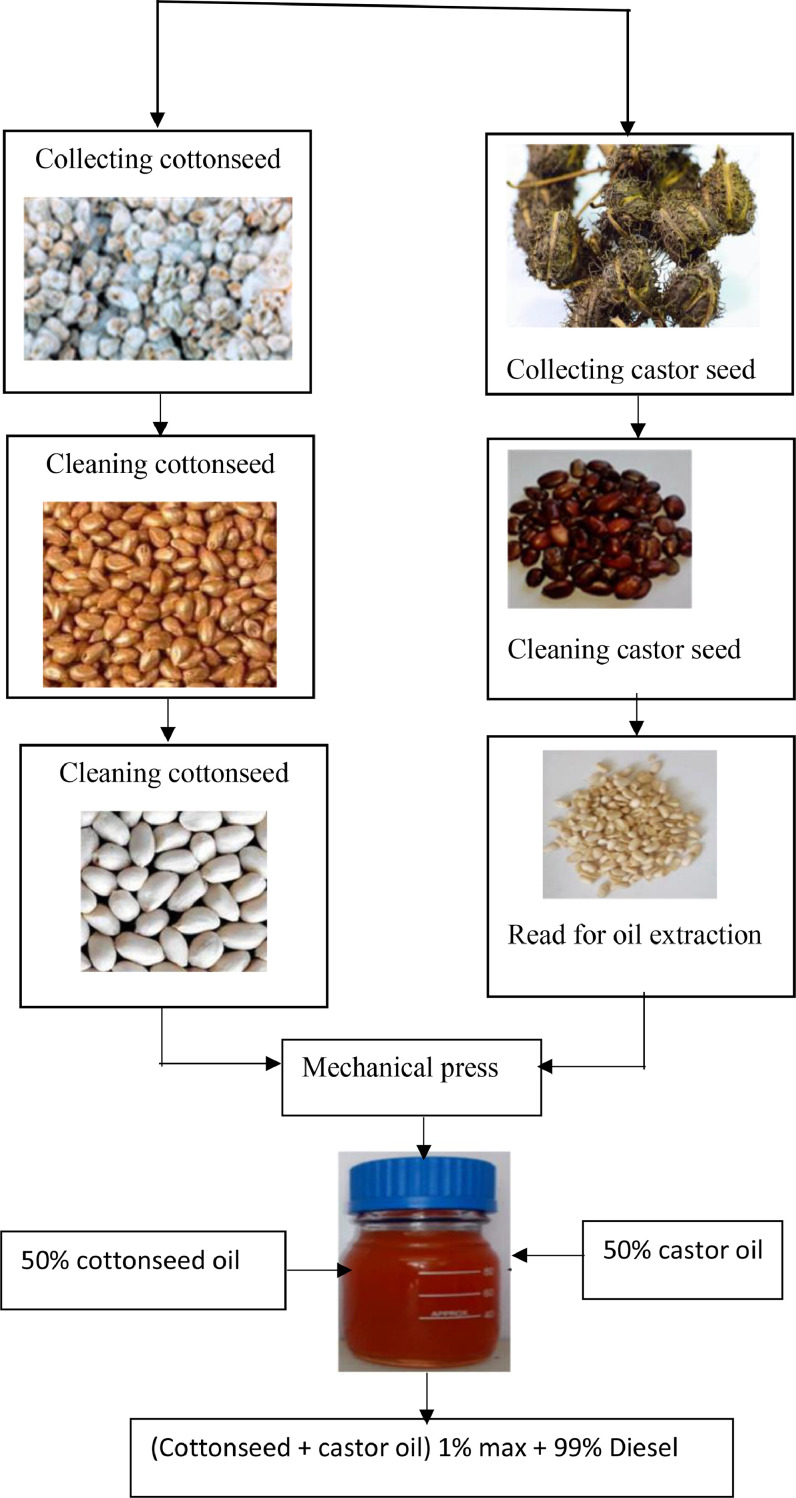
Overall extraction and blending process of Cottonseed oil and castor seed oil as additives.

The entire extraction and blending procedure for castor and cottonseed oil is depicted in Fig. 7. The same techniques used for other vegetable oils—solvent extraction, crushing, and pressing—can be used to extract cottonseed oil from plant seeds. Solvent extraction is the most widely utilized technique for commercial cottonseed oil extraction. However, the extraction method in this work uses a mechanical press. Thus, mechanical presses are only used to extract 18.92% of the oil. The percentage of yield is calculated using Eq. (1).

(1) $$\%\;of\;\;yield=\frac{\mathrm{mass}\;\mathrm{of}\;\mathrm{the}\;\mathrm{extracted}\;\mathrm{oil}}{\mathrm{intial}\;\mathrm{mass}\;\mathrm{of}\;\mathrm{the}\;\mathrm{seeds}}*100$$

Caster seed oil is extracted using the same extraction procedure. Owing to losses, the oil yields were greatly decreased when mechanical presses were used for extraction. The amount of extracted oil is calculated using Eq. (1). As a result, 23.78% of the oil extracted from castor seeds using a mechanical press.

## Limitations

This study's primary drawback is that it only addresses engine lubrication in relation to controlling sulfur concentration. Following combustion, the sulfur in diesel fuel produces sulfuric acid, which corrodes engine metal surfaces. Corrosive wear can result from surface corrosion within a dynamic system, such as the cylinder wall or liner. Similarly, the two main problems with cottonseed oils are trans fat and gossypol. Gossypol, a naturally occurring chemical present in cottonseeds, can be harmful in large amounts. However, safe levels of gossypol in cottonseed oil are ensured by modern extraction methods. Similar to other vegetable oils, cottonseed oil contains certain trans fats. Still, efforts are being made to reduce the quantity of trans fats in edible oils. Furthermore, the viscosity of castor oil is distinct. Its viscosity is 2.42 kg/m-s at 10 °C. However, over time, it tends to jam up, making it less suitable for engines that are frequently rebuilt, such as racing engines. Only a small amount—less than 1%—is utilized as an additive in this study.

## Ethics Statement

There is no animal involvement in this research.

## CRediT Author Statement

While the corresponding author originated the research proposal and provided funding for the resource, the principal author is the primary investigator. The third author edited the manuscript.

## Data Availability

Castor Oil and Characterization of Cotton and Castor Mixed Seed Oil Additives with Diesel Fuel (Original data) (Bioenergy).
